# Productivity Cost Due to Maternal Ill Health in Sri Lanka

**DOI:** 10.1371/journal.pone.0042333

**Published:** 2012-08-03

**Authors:** Suneth Agampodi, Thilini Agampodi, Nuwan Wickramasinghe, Santhushya Fernando, Umanga Chathurani, Wathsala Adhikari, Ishani Dharshika, Dhanaseela Nugegoda, Samath Dharmaratne, David Newlands

**Affiliations:** 1 Department of Community Medicine, Faculty of Medicine and Allied Sciences, Rajarata University of Sri Lanka, Saliyapura, Sri Lanka; 2 Department of Community Medicine, Faculty of Medicine, University of Peradeniya, Peradeniya, Sri Lanka; 3 Department of Economics, Aberdeen University, Scotland, United Kingdom; Tabriz University of Medical Sciences, Islamic Republic of Iran

## Abstract

**Background:**

The global impact of maternal ill health on economic productivity is estimated to be over 15 billion USD per year. Global data on productivity cost associated with maternal ill health are limited to estimations based on secondary data. Purpose of our study was to determine the productivity cost due to maternal ill health during pregnancy in Sri Lanka.

**Methods and Findings:**

We studied 466 pregnant women, aged 24 to 36 weeks, residing in Anuradhapura, Sri Lanka. A two stage cluster sampling procedure was used in a cross sectional design and all pregnant women were interviewed at clinic centers, using the culturally adapted Immpact tool kit for productivity cost assessment. Of the 466 pregnant women studied, 421 (90.3%) reported at least one ill health condition during the pregnancy period, and 353 (83.8%) of them had conditions affecting their daily life. Total incapacitation requiring another person to carry out all their routine activities was reported by 122 (26.1%) of the women. In this study sample, during the last episode of ill health, total number of days lost due to absenteeism was 3,356 (32.9% of total loss) and the days lost due to presenteeism was 6,832.8 (67.1% of the total loss). Of the 353 women with ill health conditions affecting their daily life, 280 (60%) had coping strategies to recover loss of productivity. Of the coping strategies used to recover productivity loss during maternal ill health, 76.8% (n = 215) was an intra-household adaptation, and 22.8% (n = 64) was through social networks. Loss of productivity was 28.9 days per episode of maternal ill health. The mean productivity cost due to last episode of ill health in this sample was Rs.8,444.26 (95% CI-Rs.6888.74-Rs.9999.78).

**Conclusions:**

Maternal ill health has a major impact on household productivity and economy. The major impact is due to, generally ignored minor ailments during pregnancy.

## Introduction

The leading cause of DALYs (lost) among reproductive age group women in developing countries, are due to maternal conditions [1]. Pregnancy related conditions still account for a substantial component of the global disease burden. WHO has estimated an annual loss of nearly 40 million DALYs due to maternal conditions, due to 50 million incidents of pregnancy related complications [2]. Better care for women during pregnancy could make a large contribution in reducing the global burden of pregnancy related diseases. The disease burden estimates of maternal conditions are based both on estimated and reported data. Pregnancy is a unique situation where gross under-reporting of ill-health conditions could occur. The accepted norms of “minor ailments” during pregnancy as well as other conditions could go un-noticed to the health care systems due to the traditional health seeking behaviors prevalent in the developing countries. Although most of these conditions are not life threatening, the high prevalence and incapacitation of these conditions could have a significant impact on the disease burden among pregnant women.

The global impact of maternal ill health on economic productivity is estimated to be over 15 billion USD per year [3]. In developing countries, 58%–80% of all pregnant women experience acute ill health conditions during pregnancy [4]. While some of these ill health conditions are diagnosed and treated properly, most pregnant women suffer from these conditions, limiting their daily productivity. In developing countries, loss of productivity and related cost of pregnancy has a major impact on the household economy. The costs of lost productivity because of ill health–and the costs of seeking treatment–can be offset by coping strategies [5]. Households may have to draw upon savings or borrow money or sell assets. They can also reallocate labour, usually within the household. However, all coping strategies have costs of their own which may result in lower household productivity and quality of care for children and pregnant women which in turn hinders their future productivity.

Published literature on productivity losses associated with maternal ill health conditions is scarce. In a WHO review on cost of maternal ill-health, unpublished data from Ethiopia, Mauritania, Senegal and Uganda reported an annual productivity cost due to maternal ill health ranging from $7.28 to $50.1 million [5]. Almost all productivity loss data related to maternal ill health are estimates based on secondary data. These estimates are often challenged due to wide variation of household activities and also due to lack of incorporation of loss of productivity due to reduced efficiency. In most places, loss of productivity is narrowly focused on paid work and the contribution by unemployed pregnant women in household work is often neglected. In societal point of view, the work carried out by pregnant women is equally important in household income. Amidst the many controversies on the estimation of productivity losses, the IMMPACT project, a global research initiative for the evaluation of safe motherhood intervention strategies, developed a validated field tested tool kit for productivity cost evaluation using human capital approach, which included the household productivity of pregnant women [6]. This tool kit has been validated and field tested in Ghana, and WHO recommends the use of this tool kit for evaluating cost consequences related to maternal health. Yet, no published data is available on the field application of this tool kit.

We translated and culturally adapted the ‘IMMPACT tool kit’ for productivity cost and carried out a household study to estimate the productivity losses associated with maternal ill health in Sri Lanka. Sri Lanka was considered ideal for this assessment due to several facts. In global maternal health agenda, Sri Lanka is considered as a role model for maternal mortality reduction. Present maternal mortality ratio is 34/100,000 live births and this is an early achievement of millennium development goals for the country. Sri Lanka is having an excellent maternal mortality investigation procedure with island wide coverage. It is high time for the country to move on and improve the maternal morbidity surveillance. Prioritization of morbidity surveillance needs data on disease burden and economic impact of maternal ill health, which is lacking at present. Aim of the present study to determine the productivity loss associated with the most recent episode of ill health among a sample of pregnant women from Sri Lanka.

## Results

### Study Sample

Of the selected 502 eligible pregnant women, 484 participated in the study, 18 of the questionnaires were excluded from the analysis due to missing data. Ethnic composition of the sample was Sinhalese 437 (93.8%), Moor 26 (5.6%) and Tamil 3 (.6%). Primi gravida women accounted for 40.6% (n = 189) of the study sample. Fifty percent (n = 233) of the pregnant women live in extended families. In 69.4% of the families, either the pregnant woman (n = 14, 3%) or her husband (n = 308, 66.1%) was the household head. Formal employment was reported by 12.3% (n = 57) of pregnant women. Majority (87.7%) were housewives. Table 1 shows other household characteristics of the study sample.

**Table 1:** Household characteristics of the study sample of 466 pregnant women.

Household characteristics		
Mean age of the pregnant women (SD)	27.3 yrs	(5.5 Yrs)
Median household size (IQR)	5	(3–5)
Mean monthly per capita income (SD)	$80.7	($61.1)
Mean number of hours engaged in daily activities(SD)	16 hrs	(1.2 hrs)

### Maternal Ill Health and Productivity Loss

Of the 466 pregnant women studied, 421 (90.3%) reported that they had at least one ill health condition during the pregnancy period. Of the 421, in 353 (83.8%) the most recent episodes of ill health were reported as affecting their day to day life. Hospital admission was needed for 50 (11.9%) such episodes. Total number of days spent in hospitals was 246, with an average of 4.9 days per admission. Common symptoms of the most recent episode of ill health condition included, severe nausea and vomiting (103, 24.5%), backache (n = 45, 10.7%) and cough and cold (n = 42, 10.0%) (Table 2).

**Table 2:** Leading causes of ill health and the productivity loss during the most recent episode of ill health by main symptom among 353 Sri Lankan pregnant women who reported ill health conditions affecting daily work.

Main symptoms of the lastepisode of illness	Number of episodes (%)		Days lost (Unadjusted)					
			Median (IQR)		Mean (SD)		Total (%)	
Nausea and vomiting	94	(26.6)	36	(18–63)	44.5	(34.9)	4186.6	(41.1)
Backache/pelvic pain	59	(16.7)	15	(5.6–36)	32.9	(41.8)	1942.4	(19.1)
Cramps	29	(8.2)	12	(5.6–24)	27.8	(38.7)	805.2	(7.9)
Heartburn/regurgitation	23	(6.5)	24	(12–51)	31.2	(28.6)	717.6	(7.0)
Headache	20	(5.7)	7.8	(1.8–29)	23.4	(31.7)	467.8	(4.6)
Cough and cold	12	(3.4)	4.8	(1.8–12)	9.3	(11.4)	315.4	(3.1)
Vertigo	34	(9.6)	12	(2.8–27)	20.3	(27.5)	304.6	(3.0)
Varicose veins	15	(4.2)	27	(21–36)	34.1	(26.6)	273	(2.7)
Fever	8	(2.3)	6.2	(3.6–12.4)	15.3	(21.9)	230.2	(2.3)
Candidiasis	15	(4.2)	21	(11–28)	19.9	(11.3)	159	(1.6)
Swollen legs	8	(2.3)	24	(6–30)	19.2	(12.6)	134.2	(1.3)
Vaginal bleedings	7	(2.0)	14	(13–20)	22.8	(25.2)	113.8	(1.1)
Tiredness	5	(1.4)	6.5	(0.8–27.5)	13.3	(15.3)	106	(1.0)
Shortness of breath	8	(2.3)	5.1	(1.2–8.6)	5.4	(4.5)	32.2	(0.3)
Dysuria	6	(1.7)	2.8	(1–6)	3.8	(3.4)	26.4	(0.3)
Raised blood pressure	7	(2.0)	8.4	(2.8–12.8)	8.0	(5.0)	24	(0.2)
Other symptoms	3	(0.8)	12	(2.8–49)	29.2	(37.0)	350.4	(3.4)
Total	353	(100)	18	(5–36)	28.9	(33.6)	10,188.8	(100)

Median duration was calculated because the distribution was skewed to right. Mean values are also presented for further analysis.

### Productivity Loss during the Last Episode of Ill Health

Total incapacitation requiring another person to carry out all their routine activities during the last episode of ill health was reported by 122 (26.1%) women. In this study sample, during the last episode of ill health, total number of days lost due to absenteeism was 3,356 (32.9% of total loss) and the days lost due to presenteeism was 6,832.8 (67.1% of the total loss) (Table 3). Loss of productivity due to hospital admission (246 days), which was included in the absenteeism, was only 2.4% of the total loss.

**Table 3:** Loss of productivity during the most recent episode of ill health among 353 Sri Lankan pregnant women who reported ill health conditions affecting their daily work.

Productivity loss	Mean (SD)		Median (IQR)		Total
Days lost due to absenteeism	9.5	(23.7)	0	(0–4)	3,356.0
Days lost due to presenteeism	19.4	(23.0)	12	(2–24.4)	6,832.8
Total days lost	28.9	(33.6)	18	(5–36)	10,188.8
Days recovered	15.1	(20.4)	6	(.4–24)	5,329.8
Adjusted total loss	13.8	(22.4)	4.2	(0–18)	4,859.0

Of the 353 women with ill health conditions affecting daily life, 280 (60%) had coping strategies to recover loss of productivity. Of the coping strategies used to recover productivity loss during maternal ill health, 76.8% (n = 215) was an intra-household adaptation (a household member carrying out pregnant woman’s’ work), and 22.8% (n = 64) involved social networks (neighbor, friend or relative helped in day to day work of pregnant woman). Total days recovered through coping strategies were 5,329.8 (Table 3). The adjusted total loss was 4,859 days with an average of 13.8 days per episode among the women who reported ill-health conditions, affecting daily life.

Based on these figures, estimated loss of productivity due to the most recent episode of ill health condition, among currently pregnant women was 28.9 days per single episode of ill health.

### Leading Causes of Productivity Loss

The leading cause of productivity loss (Table 2) in this study sample was nausea and vomiting associated with pregnancy. It accounted for 41.1% of total productivity loss and the median number of days lost was 36 (IQ range 18–63). Backache and pelvic pain was the second leading cause of productivity loss followed by leg pains and cramps. Though cough and cold was reported as the third leading ill health condition (10%), productivity loss due to this condition was only 3.1%.

### Productivity Cost of Maternal Ill Health during Pregnancy

The mean productivity cost due to the last episode of ill health among 353 Sri Lankan pregnant women in this sample was Rs.8,444.26 (SEM Rs. 791.08). After adjustment for copying strategies the cost of a single episode of ill health during pregnancy was Rs.3,925.51 (SEM Rs. 481.31). The total cost for the last episode of ill health among this study sample was Rs.2,854,160 and the adjusted total cost was Rs.1,350, 375.

## Discussion

In the present study, loss of productivity due to the most recent episode of ill health condition, among currently pregnant women was estimated to be 28.9 days per episode of ill health condition. The productivity cost of a single episode of ill health was Rs.8,444.26 (95% CI-Rs.6888.74-Rs.9999.78). Further, the present study revealed that traditionally ignored “minor ailments during pregnancy” accounted for most of the productivity losses. Hospitalizations accounted only for 2.4% of the total productivity loss. Of the total loss, 67.1% was due to presenteeism. These findings show that the productivity losses associated with maternal ill health are substantial. It also shows gross underestimation of the burden of maternal morbidities and the error of estimating the burden of maternal ill health based only on hospitalized patients. Findings confirm previous observations of the profound effect of presenteeism on productivity loss [7].

Limitations and possible biases that could have an effect on these findings should be taken into account, before interpreting these data. Observation of predominant productivity loss due to “minor” conditions could partly be due to several sources of selection bias. Pregnant women with severe maternal ill health conditions who were in hospitals at the time of the study could not be included in the study. Further, women who had severe ill health conditions during the last episode of ill health, and ended up with pregnancy losses were also not included in the study sample, as currently pregnant women. We also excluded pregnant women with a gestational age more than 36 weeks, during which most severe perinatal morbidities are common. Generalization of findings should not include the total antenatal period. Nevertheless, studies done on health related productivity losses usually report higher levels of productivity losses due to prevalent minor conditions [8], than less prevalent severe conditions. However these biases could have resulted in underestimation of the true value of the productivity loss, and the estimate calculated in this study could be an underestimation of the true picture.

In this study sample, 41. 1% of total productivity loss was due to nausea and vomiting associated with pregnancy (NVP). This condition may have included hyperemisis gravidarum, which we did not attempt to differentiate. Extensive evidence is available in published literature on the burden of disease of NVP [9] and its’ effect on life limitations [10] and psychological impairment [11], [12], [13], [14]. It has been shown that NVP incurs substantial economic burden [15], [16] which strengthen the finding of our study. Despite having evidence on its burden and effect, control and prevention of NVP is difficult, because the optimal target for treatment and the effect of potential treatment on the fetus is still unknown. Present treatment guidelines are suboptimal and clear guidelines for management of NVP is needed to tackle this disease burden and productivity loss [17]. As the fourth leading cause of productivity loss (heartburn and regurgitation) in this study is also related to NVP [18] a combined approach should help to prevent nearly half of the productivity losses.

It is found that other leading causes of productivity loss such as backache, pelvic pain, oedema and varicose veins are often not treated in the ideal manner. It is important to emphasize the socio-cultural and behavioral component on both health seeking and physicians concern on these conditions. Our focus group discussions revealed that these listed minor ailments are considered as a “normal” (by community) or “physiological” (by health professionals). Studies on backache shows non pharmaceutical interventions are more effective than commonly used pharmaceutical management [19]. For varicose veins and edema, commonly used external compression stockings/bandages are not helpful, limited evidence suggests rutoside and reflexology are helpful [20], however further studies are needed for general recommendations to be made.

Even though the IMMPACT tool is using coping strategies to calculate adjusted productivity loss, these are not costless. It is debatable whether these are coping strategies should be included as coping strategies or not, in relation to productivity cost, because the coping is at the cost of another persons’ productivity. Since the recovery of productivity loss of pregnant woman is mostly through intra-household strategies, one may argue that it affects the household productivity function. This fact is open for discussion, as the present paper is the first of its’ kind among pregnant women.

In conclusion, this study provides strong evidence to show that the traditionally ignored “minor ailments” are the leading causes of productivity loss in pregnancy (excluding perinatal conditions). We also showed that maternal ill health could have a major impact on household economy through the loss of productivity of pregnant women. Though poverty has not been shown to be associated with productivity loss, effect of this loss could be devastating in poor households. Control and prevention of identified major causes of productivity losses should be included in maternal health programmes, in order to make maternal healthcare to take a more holistic approach. Further studies are needed to identify underlying causes and variations in productivity losses during pregnancy across cultures.

## Methods

Reporting of this paper is according to the STROBE requirements.

We conducted a cross sectional descriptive study to determine the productivity cost due to maternal ill health.

### Study Setting

Present study was carried out in the Anuradhapura district, located in the north central part of Sri Lanka. The total population residing in the Anuradhapura district in 2010 was 886,945, and of them, 92.7% live in the rural sector. Annually, around 19,000 pregnant women are registered at antenatal clinics in the Anuradhapura district which has 19 public health divisions, known as Medical Officer of Health (MOH) areas. Each MOH area is divided into sub-divisions called Public Health Midwife (PHM) areas with a population ranging from 1,500 to 3,000 in each area and the maternal health services are provided through the area PHM. Registration of pregnant women by PHMs is reported as nearly 100% in this district.

### Participants

The study population included all pregnant women with gestational ages which ranged from 24 to 36 weeks, and residing in the Anuradhapura district. Sample was selected from those who registered in the field antenatal clinics. In this first field application of the Sinhalese version of IMMPACT tool kit, we selected this specific group to exclude perinatal conditions, which need to be investigated separately. A two stage cluster sampling procedure was used to recruit pregnant women to this study. In the first stage, five MOH areas were selected purposely to represent different geographical areas of the Anuradhapura district. In the second stage, eligible pregnant women within the specified gestational ages were selected using pregnant women registers available in the PHMs offices of the selected MOH areas. All eligible pregnant women were invited to participate in the study by PHMs. Pregnant women who consented to participate in the study, were interviewed at clinic centers or health centers, where investigations for pregnant women were carried out. Medical graduates collected data from all pregnant women using an interviewer administered questionnaire which included IMMPACT tool kit. Prior to data collection, all data collectors were given a comprehensive training on data collection procedures, use of protocols, probing and extracting data from records. During the pilot study and pre-test, all issues related to data collection data quality, and data incompatibility, were discussed.

### Study Size

In India, a community based study showed that the percentage of pregnant women experiencing morbid conditions during the antenatal period ranged from 61–95% [21]. We hypothesized that in our population at least 70% of pregnant women would experience acute ill-health conditions during pregnancy. Sample size was calculated to detect at least 70% of the ill-health conditions for a population of 15,000 deliveries, with 10% relative precision and 95% confidence limits. With a 1.5 design effect for cluster design, required sample size for the present study was estimated as 476 pregnant women.

### Study Tool

The primary data collection tool in this study was IMMPACT productivity cost tool. The IMMPACT productivity tool is designed to collect data on women’s work activities and labour productivity outcomes during pregnancy and the puerperium. The productivity loss estimation is based on the most recent episode of maternal-ill health. As behavioral responses and consequences of ill health are dependent on other household-wide characteristics, the questionnaire also collected data on various socio-economic aspects of the household.

The questionnaire asked respondents to estimate the time they were completely unable to work, defined as carrying out their normal daily activities. This is termed absenteeism. Respondents were also asked to estimate their reduced effectiveness of working while ill. This is termed presenteeism and is the product of time spent working while unwell and an estimate of how much less effectively the respondent is working. The sum of absenteeism and presenteeism is a measure of the productivity costs of ill health prior to adjustment for coping strategies. The questionnaire asked respondents to estimate the time received in assistance from family members and others and the effectiveness of assistance received. Deduction of this measure from the sum of absenteeism and presenteeism gives an estimate of productivity costs, after adjustment for coping strategies. This tool measures the length of the period during which losses are experienced and the extent of the production loss. The latter is assessed by use of a visual analogue scale (VAS) in which respondents were asked to estimate their average efficiency on days when they were ill on a scale of 0 (“illness did not permit me to work”) to 5 (“illness did not affect my work”). Same type of VAS was used to calculate the recovery of losses through coping strategies.

Even though this tool kit was developed and validated in a field study in Ghana, published literature is not available for use of the tool. All available information on IMMPACT tool are from the IMMPACT web site (http://www.immpact-international.org/).

### Translation, Cultural Adaptation, and Validation of IMMPACT Tool Kit

IMMPACT questionnaire was introduced to the team of investigators by the Principal Investigator and the concept, the utility and the application procedure was discussed. A rigorous procedure of cultural adaptation was used. In-depth interviews, informal discussions and focus group discussions were conducted to identify similar local variables used in the original questionnaire and include local terminology. After analysis of qualitative data, we used the cultural adaptation procedure suggested by Murray and Sumathipala [22], followed by two rounds of field testing. Field testing was carried out for initial validation of the culturally adapted tool kit. The questionnaire was evaluated by an investigator from the original IMMPACT design team during and after the field test, to confirm that the construct validity of the original questionnaire was not altered, during the translation and the cultural adaptation process. Face validity and consensual validity was assessed by a health economist, a community physician, epidemiologist and a maternal and child health specialist. As a part of our main study, we analyzed data for validity and reliability. A high concurrent validity was shown for rank ordered data (Spearman’s r ranged from .891 to .903) related to pregnancy and socio-demographic details collected through the productivity cost tool. Final version of the Productivity cost questionnaire (English and Sinhalese versions) is available for researchers on request.

To minimize the recall bias, which is inheriting to all cross sectional studies and specially this tool, where the last episode of ill health could be more than three months back, we used available medical records, notes in antenatal record and clinic books. All participants were asked to bring these documents to the interview. The questionnaire itself was about episodes that limited day to day work, which is minimally affected by the recall bias.

### Data Analysis

For the purpose of this study we defined the productivity loss in terms of days lost and the productivity cost in monitory terms by translating the days lost using household income data. Productivity loss calculation was carried out as described in ‘IMMPACT tool kit’ [6]. Days lost were calculated by the sum of absenteesm and presenteesm. Presenteesm was calculated using the efficiency of work as measured in VAS and duration of work with reduced efficiency. Productivity loss was then converted to cost, based on the per capita income, assuming that work carried out by the pregnant women is equally important in contributing to the household income. Per capita income data collected from household productivity and economic activities sections of the IMMPACT tool kit were used for this cost estimation. We have given equal weight to children in per capita income calculation, after extensive panel discussin on local household economics.

### Ethical Considerations

Ethical clearance for the present study was obtained under the study on “Disease burden and economic impact of maternal morbidity”, from the Ethical Review Board of the Faculty of Medicine and Allied Sciences, Rajarata University of Sri Lanka. Informed written consent was obtained from all pregnant women prior to data collection.
